# Effect of dietary phosphorus intake and age on intestinal phosphorus absorption efficiency and phosphorus balance in male rats

**DOI:** 10.1371/journal.pone.0207601

**Published:** 2018-11-19

**Authors:** Colby J. Vorland, Pamela J. Lachcik, Loretta O. Aromeh, Sharon M. Moe, Neal X. Chen, Kathleen M. Hill Gallant

**Affiliations:** 1 Department of Nutrition Science, Purdue University, West Lafayette, IN, United States of America; 2 Division of Nephrology, Department of Medicine, Indiana University School of Medicine, Indianapolis, IN, United States of America; 3 Department of Anatomy and Cell Biology, Indiana University School of Medicine, Indianapolis, IN, United States of America; 4 Department of Medicine, Roudebush Veterans Affairs Medicine Center, Indianapolis, IN, United States of America; The University of Manchester, UNITED KINGDOM

## Abstract

Intestinal phosphorus absorption is an important component of whole-body phosphorus metabolism, and limiting dietary phosphorus absorption is particularly of interest as a therapeutic target in patients with chronic kidney disease to manage mineral bone disorders. Yet, mechanisms and regulation of intestinal phosphorus absorption have not been adequately studied and discrepancies in findings exist based on the absorption assessment technique used. *In vitro* techniques show rather consistent effects of dietary phosphorus intake level and age on intestinal sodium-dependent phosphate transport. But, the few studies that have used *in vivo* techniques conflict with these *in vitro* studies. Therefore, we aimed to investigate the effects of dietary phosphorus intake level on phosphorus absorption using the *in situ* ligated loop technique in three different aged rats. Male Sprague-Dawley rats (n = 72), were studied at 10-, 20-, and 30-weeks-of-age on a low (0.1%), normal (0.6%), or high (1.2%) phosphorus diet in a 3x3 factorial design (n = 8/group). Rats were fed their assigned diet for 2-weeks prior to absorption testing by jejunal ligated loop as a non-survival procedure, utilizing ^33^P radioisotope. Metabolic cages were used for determination of calcium and phosphorus balance over the final four days prior to sacrifice, and blood was collected at the time of sacrifice for biochemistries. Our results show that phosphorus absorption was higher in 10-week-old rats compared with 20- and 30-week-olds and this corresponded to higher gene expression of the major phosphate transporter, NaPi-2b, as well as higher whole-body phosphorus balance and net phosphorus absorption. Dietary phosphorus intake level did not affect jejunal phosphorus absorption or NaPi-2b gene expression. Our results contrast with studies utilizing *in vitro* techniques, but corroborate results of other rodent studies utilizing *in situ* or *in vivo* methods. Thus, there is need for additional studies that employ more physiological methods of phosphorus absorption assessment.

## Introduction

Phosphorus is an essential nutrient for normal physiological function. However, elevated serum phosphorus has been linked to increased cardiovascular disease [1], bone disease [2], and mortality [3, 4]. This is particularly true for patients with chronic kidney disease (CKD) [5], where the failing kidney has a reduced capacity for renal excretion. In normal physiology, the kidney is the primary site of regulation for phosphate homeostasis [6]. Thus, as the kidneys fail, therapeutic options focus on reducing intestinal phosphorus absorption through dietary restriction, luminal phosphate binding, or inhibiting intestinal phosphorus transport. However, mechanisms and regulation of intestinal phosphorus absorption have not been adequately studied, especially when compared to that in the kidney. Renal phosphate reabsorption is nearly completely transcellular and sodium-dependent and is regulated by the major known phosphaturic hormones, parathyroid hormone (PTH) and fibroblast growth factor 23 (FGF23) [7]. In contrast to the kidney, intestinal phosphorus absorption occurs by both sodium-dependent and sodium-independent pathways [8]. However, the relative importance of each component is debated.

The majority of the existing literature on phosphorus absorption has relied on *in vitro* methods of absorption assessment including brush border vesicles or Ussing chambers. Low phosphorus diets have been shown to increase sodium-dependent intestinal phosphate uptake when measured by the rapid-filtration technique in isolated brush border membrane vesicles (BBMV) from healthy rats [9–12] and mice [13–16], and by Ussing chamber in rat [17] and pig [18]. In contrast to in vitro studies, the limited in vivo studies in rodents have conflicting results with low phosphate diet both increasing [19] and having no effect on [20] intestinal phosphate absorption. Possible reasons for this discrepancy include the intestinal region tested (duodenum vs jejunum), sex and species of the animals (female Wistar vs male Sprague-Dawley), normal vs uremic animals, age of animals studied, differences in the technique used, and the duration of study. Given the importance of understanding precise mechanisms of intestinal phosphorus absorption *in vivo* in order to design more effective therapeutic interventions for patients with CKD, we studied rats at three ages with low, moderate, and high phosphorus diets. Since the majority of prior studies on intestinal phosphorus transport have utilized *in vitro* and *ex vivo* methods with results at odds with the limited *in situ/in vivo* results available, we selected the *in situ* intestinal ligated loop method to assess phosphorus absorption by a more physiologic technique. Our findings show that intestinal phosphorus absorption by this method is affected by age, but is not affected by dietary phosphorus intake level, which conflicts with prior *in vitro* studies, but largely corroborates the limited prior *in situ/in vivo* studies.

## Materials and methods

### Animals

This was a 3x3 factorial design study. Seventy-two male Sprague-Dawley rats (Harlan Laboratories, Indianapolis, IN) were randomly assigned to 10-, 20-, or 30-week-old age groups, and randomly assigned to low, normal, or high dietary phosphorus within each age group (n = 8 rats/age x diet group). Rats were fed standard rat chow containing 0.7% phosphorus and 1.0% Ca (Harlan Teklad 2018, Indianapolis, IN) and water *ad libitum* until 8-, 18-, or 28-weeks of age (for the 10, 20, and 30-week-old age groups, respectively), at which time they were switched to their assigned study diets and fed *ad libitum* for two weeks prior to sacrifice. The low-phosphorus (LP), normal-phosphorus (NP), and high-phosphorus (HP) diets contained 0.1, 0.6, and 1.2% phosphorus, respectively, all with 0.6% Ca (Harlan Teklad, Indianapolis, IN: TD.85010, LP, TD.84122, NP, TD.85349, HP). Rats were housed individually in wire-bottom metabolic cages for the two weeks of the assigned diet period, and phosphorus and calcium balance were performed during the last four days prior to sacrifice. Body weights were taken weekly. The light-dark cycle was maintained from 6AM-6PM. This protocol was approved by the Purdue University Animal Care and Use Committee (Protocol Number: 1402001030).

### Intestinal phosphorus absorption efficiency

Intestinal phosphorus absorption efficiency was determined by *in situ* jejunal ligated loop absorption tests performed as a non-survival procedure before sacrifice. On day 14 of the assigned diet, rats were fasted for three hours prior to the ligated loop absorption test. Groups were order-balanced for treatment and testing to average the potential time-effect on absorption [21]. The absorption test protocol was based on that published by Marks et al. [20], with the exception of the anesthetic. The rats were anesthetized by inhalation of isoflurane and kept warm with a heating blanket during the non-survival procedure for approximately 45 minutes. A jugular vein catheter was placed for blood sampling, and a baseline blood sample (0.4 mL) was collected. The abdominal cavity was opened, and two ligatures were placed to create a ~5 cm segment of the jejunum. The first ligature was placed approximately 1 cm distal to the suspensory muscle of the duodenum (Ligament of Treitz) and firmly tied twice. The second ligature was loosely tied ~5 cm distal to the first ligature. Transport buffer (0.5 mL) containing (mmol/L) 16 Na-N-2-hydroxyethylpiperazine-N0-2-ethanesulfonicacid, 140 NaCl, 3.5 KCl, 0.1 KH2PO4, and ~5 uCi ^33^P (^33^P-orthophosphoric acid, PerkinElmer, Waltham, MA) was injected by gastight syringe (Hamilton, Reno, NV) into the jejunal lumen through the distal ligature, which was immediately tied off following the injection of the radioactive transport buffer. Blood (0.4 mL/sampling) was collected at 5, 10, 15, and 30 min post-injection in lithium heparin tubes and centrifuged at 10,500 g for 10 minutes (Micro 18R, VWR, Radnor, PA) to separate plasma. Immediately after the final 30-minute post-injection blood draw, the jejunal loop was removed, measured for length, and placed in a 20mL scintillation vial containing 6 mL Soluene-350 (PerkinElmer, Waltham, MA) for digestion in preparation for liquid scintillation counting of ^33^P activity remaining in the jejunal loop. After heating overnight at 45°C in an oven, the dissolved jejunal loop was split into two vials and lightened with 0.6 mL 30% hydrogen peroxide (Avantor, Center Valley, PA) to reduce color quench.

Under anesthesia, rats were sacrificed by cardiac puncture and exsanguination followed by cardiac excision. Blood (0.5 mL) was aliquoted into lithium heparin tubes for hematocrit measurement and stored on ice until centrifugation for 5 minutes at 5,900 g (Readacrit, Clay Adams, Parsippany, N.J.). Remaining blood was aliquoted into lithium heparin tubes for plasma separation as described above, flash frozen in liquid nitrogen and stored at -80C until analysis.

Liquid scintillation counting of the ^33^P transport buffer solution, plasma samples from each time point, and digested jejunal loop was performed on a Tri-Carb 2910 TR Liquid Scintillation Analyzer (PerkinElmer, Waltham, MA). 500 uL of transport buffer solution and 250 uL plasma samples were counted in 15 mL of EcoLite liquid scintillation cocktail (MP Biomedicals, Santa Ana, CA), and digested loops were counted in 17 mL of Hionic-Fluor scintillation cocktail (PerkinElmer, Waltham, MA). Appropriate quench curves for each scintillation cocktail were used to adjust counts per minute to disintegrations per minute [22] (**S1 File**). Absorption of ^33^P was evaluated two ways: 1) area under the curve (AUC) was calculated for plasma ^33^P activity over 30 minutes, and 2) percent intestinal phosphorus absorption efficiency over 30 minutes was calculated as:

1 − (^33^P activity remaining in digested loop) / (Total ^33^P activity in 0.5mL dose) ∙ 100

### Phosphorus and calcium balance and net absorption

On days 10 through 14 of the assigned study diet, all urine and feces were collected and diet weighed daily to assess 4-day average phosphorus and calcium balance and net absorption. Feces and diet were ashed in a muffle furnace (Thermolyne Sybron Type 30400, Dubuque, IA) for 10 days at 550°C. Feces were then diluted 140X and diet 14X with 2% nitric acid. Urine was diluted 11X with 2% nitric acid. Phosphorus and calcium in urine, feces, and diet were quantified by inductively coupled plasma-optical emission spectrophotometry (ICP-OES; Optima 4300DV, Perkin Elmer, Shelton, CT). Urine creatinine was determined by colorimetric method using a COBAS Integra 400 Plus (Roche Diagnostics, Indianapolis, IN). Four-day phosphorus balance was calculated as dietary phosphorus intake (mg/d) minus urine and fecal phosphorus excretion (mg/d), and net phosphorus absorption as phosphorus intake (mg/d) minus fecal excretion (mg/d). Calcium balance and net calcium absorption were calculated similarly.

### Intestinal phosphate transporter gene expression

After the completion of the ligated loop absorption tests and the removal of the radioactive jejunal loop, approximately 5 cm of jejunum distal to the loop and 5 cm of duodenum distal to the pylorus were removed, cut open and rinsed with ice-cold deionized water. The mucosal layers were scraped, and mucosa from each intestinal segment was placed into TRI Reagent (Fisher Scientific, Hampton, NH) and flash frozen in liquid nitrogen for later mRNA quantification by RT-PCR.

Total RNA was extracted using Omega HP Total RNA Kit (R6812-00, Omega Bio-tek, Norcross, GA; modified to add a chloroform extraction step). Concentration and purity were determined on a NanoDrop 2000c spectrophotometer at 260, 280, and 230nm (Thermo Fisher Scientific, Waltham, MA). Real-time PCR amplifications were performed using TaqMan gene expression assays (TaqMan MGP probes, FAM dye-labeling) with Applied Biosystems ViiA 7 Real-Time PCR systems (Applied Biosystems). NaPi2b (Slc34a2), PiT1 (Slc20a1), and Rplp0 primers were obtained from Applied Biosystems (Rn00584515_m1, Rn00579811_m1, and Rn03302271_gH). The ΔΔCT method was used to analyze the relative change in gene expression normalized to the housekeeping gene Rplp0.

### Plasma biochemistries

Plasma stored at -80C was thawed and analyzed for phosphorus, calcium, and creatinine concentration by colorimetric method using a COBAS Integra 400 Plus (Roche Diagnostics, Indianapolis, IN). Blood urea nitrogen (BUN), calcium and phosphorus were measured by colorimetric assay (BioAssay Systems, Hayward, CA and Point Scientific, Canton, MI). Intact parathyroid hormone (iPTH), intact fibroblast growth factor 23 (iFGF23), c-terminal (includes c-terminal fragments and intact protein) FGF23 (cFGF23) (Immutopics, San Clemente, CA), and 1,25(OH)2D3 by enzyme immunosorbent assay (Immunodiagnostic Systems, The Boldons, UK).

### Statistics

A sample size of n = 8 rats/group was determined to be sufficient to detect a 30% difference between groups for phosphorus absorption (β = 0.80, α = 0.05) based on means and standard deviations reported by Marks et al. [20]. Two-way ANOVA was performed for all outcomes with main effects for diet and age and their interaction utilizing least-squares means with Tukey post-hoc comparisons. Statistical significance was set at α < 0.05. Statistical Analysis Software version 9.3 (SAS Institute, Cary, NC) was used for all statistical analysis. Results and figures are reported as mean ± SEM of each age group on the three diets, or vice versa, unless otherwise indicated.

## Results

Body weight prior to starting the assigned diet was higher for older rats, as physiologically expected (10-week-olds: 275.8 ± 3.1 g, 20-week-olds: 425.5 ± 5.5 g, 30-week-olds: 461.5 ± 5.5 g, p < 0.0001 for all comparisons, but was not different between the diet groups, nor was there an age x diet interaction (diet main effect, p = 0.3751; age x diet interaction, p = 0.2762). At sacrifice, body weight followed a similar pattern (10-week-olds: 306.7 ± 5.8 g, 20-week-olds: 438.7 ± 4.5 g, 30-week-olds: 485.8 ± 5.7 g, p < 0.0001 for all comparisons; diet main effect, p = 0.1344; age x diet interaction, p = 0.0741).

At the time of sacrifice, plasma creatinine tended to be higher in 10-week-old rats versus 20- and 30-week-olds (0.49 ± 0.02 mg/dL vs 0.44 ± 0.01 mg/dL and 0.45 ± 0.01 mg/dL) but no post-hoc comparisons were significant due to a marginal interaction with diet (**Table 1**). Urinary creatinine increased at each age group (7.2 ± 0.2, 11.9 ± 0.4, 13.5 ± 0.4 for 10, 20, and 30-week-old, respectively (p < 0.0001 for 10 vs 20 and 30, p = 0.0042 for 20 vs 30). (**Table 1**). Creatinine clearance was lower in 10-week-old rats versus 20- and 30-week-old rats (3.6 ± 0.1 mL/min vs 4.5 ± 0.2 mL/min and 4.5 ± 0.1 mL/min, p = 0.0005 and p = 0.0003, respectively) (**Table 1**). Plasma BUN progressively declined with age but was within normal physiologic range (22.6 ± 0.5 mg/dL, 20.4 ± 0.6 mg/dL, and 18.3 ± 0.5 mg/dL for 10-, 20-, and 30-week groups respectively (p < 0.015 for all comparisons) and did not change with level of phosphorus in the diet (p = 0.0771). Plasma phosphorus was higher in 10-week old rats compared to both the 20- and 30-week olds (9.4 ± 0.3 mg/dL vs 7.5 mg/dL ± 0.2 and 7.2 ± 0.1 mg/dL, p < 0.0001 for both), but there was no difference between diets (**Table 1**). There was a significant age x diet interaction for plasma calcium, where 10-week olds had higher plasma calcium on LP compared with NP and HP (p = 0.0011 and p = 0.0005 respectively), but there were no other significant group comparisons (**Table 1**). Overall, dietary phosphorus level and age caused anticipated changes in the phosphorus-regulating hormones of 1,25D, FGF23, and PTH. There was a significant age x diet interaction for plasma iPTH where LP resulted in lower iPTH compared with NP and HP, but the magnitude was greatest in the 10-week old rats (**Table 1**). Both iFGF23 and cFGF23 were lower in the LP vs NP and HP (iFGF23: 106.6 ± 11.3 pg/mL vs 327.1 ± 14.7 pg/mL and 347.3 ± 11.8 pg/mL, p < 0.0001 for both; cFGF23: 266.6 ± 15.1 pg/mL ± vs 496.8 ± 16.6 pg/mL and 514.8 ± 21.4 pg/mL, p < 0.0001 for both) (**Table 1**). iFGF23 was also lower in 10-week-old vs 20-week-old rats (240.7 ± 27.7 pg/mL vs 282.5 ± 25.6 pg/mL, p = 0.0491) (**Table 1**). 1,25D was higher in 10-week old rats compared to 20- and 30-week (446.0 ± 19.9 pg/mL vs 339.9 ± 16.7 pg/mL and 325.1 ± 16.6 pg/mL, p = 0.0001 and p < 0.0001) and it was higher on LP vs NP (414.1 ± 24.7 pg/mL vs 328.7 ± 18.0 pg/mL; p = 0.0017), HP was not significantly different from LP (370.1 ± 16.9 pg/mL vs 414.1 ± 24.7 pg/mL, p = 0.11) or NP (370.1 ± 18.0 pg/mL vs 328.7 pg/mL, p = 0.24) (**Table 1**).

**Table 1:** Final blood and urine biochemistries†.

	10 weeks old			20 weeks old			30 weeks old			P-Values			
	LP	NP	HP	LP	NP	HP	LP	NP	HP	Model	Age	Diet	Age x Diet
**Plasma P (mg/dL)^a^**	9.2 (0.6)	9.0 (0.2)	9.9 (0.5)	7.1 (0.3)	8.0 (0.2)	7.4 (0.3)	7.2 (0.3)	7.2 (0.2)	7.2 (0.2)	**<0.0001**	**< 0.0001**	0.4715	0.1703
**Plasma Ca (mg/dL)^a^**	10.8 (0.4)	9.4 (0.1)	9.3 (0.1)	9.6 (0.2)	9.1 (0.2)	9.3 (0.2)	9.6 (0.2)	9.7 (0.1)	9.8 (0.1)	**<0.0001**	**0.0084**	**0.0004**	**0.0009**
**Plasma Creatinine (mg/dL)^a^**	0.5 (0.04)	0.5 (0.02)	0.5 (0.04)	0.5 (0.02)	0.5 (0.02)	0.4 (0.02)	0.5 (0.01)	0.5 (0.02)	0.4 (0.0)	**0.0142**	**0.0438**	0.1500	0.0504
**Urine Creatinine (mg/dL)**	6.6 (0.4)	8.0 (0.5)	7.2 (0.1)	12.4 (0.8)	10.7 (0.7)	12.5 (0.7)	13.3 (0.7)	14.3 (0.4)	12.8 (0.7)	**<0.0001**	**< 0.0001**	0.8948	0.0317
**Hematocrit (%)^b^**	42.2 (1.1)	43.1 (0.7)	44.3 (0.5)	44.6 (1.0)	45.3 (0.7)	44.1 (1.4)	44.3 (0.1)	43.9 (1.0)	45.5 (0.7)	0.2573	0.0828	0.4396	0.4635
**Creatinine Clearance (mL/min)^a^**	3.2 (0.2)	4.0 (0.2)	3.5 (0.2)	4.5 (0.4)	4.0 (0.3)	5.0 (0.3)	4.3 (0.3)	4.2 (0.2)	4.9 (0.2)	**0.0002**	**<0.0001**	0.0814	0.0641
**BUN (mg/dL)^c^**	23.6 (0.9)	21.8 (0.8)	22.5 (0.7)	22.0 (0.8)	21.0 (0.9)	18.4 (1.2)	18.7 (0.7)	18.0 (0.9)	18.3 (1.3)	**<0.0001**	**<0.0001**	0.0771	0.2731
**Plasma PTH (pg/mL)**	92.1 (28.9)	617.1 (43.7)	778.6 (64.5)	194.6 (37.9)	446.7 (40.6)	376.6 (54.9)	262.8 (62.2)	437.2 (95.6)	421.6 (49.0)	**<0.0001**	**0.0027**	**<0.0001**	**<0.0001**
**Plasma iFGF23 (pg/mL)^d^**	60.2 (4.0)	300.9 (19.1)	338.3 (24.9)	133.0 (21.1)	340.0 (32.9)	355.8 (21.0)	124.2 (16.1)	340.4 (23.5)	347.8 (16.8)	**<0.0001**	**0.0377**	**<0.0001**	0.7148
**Plasma cFGF23 (pg/mL)^d^**	292.3 (34.0)	504.0 (19.2)	554.4 (48.6)	240.8 (13.3)	458.5 (37.2)	497.6 (39.8)	266.6 (21.6)	528.0 (24.9)	492.3 (13.1)	**<0.0001**	0.1426	**<0.0001**	0.6751
**Plasma 1,25D (pg/mL)^e^**	527.7 (30.1)	404.0 (25.7)	411.3 (29.5)	385.2 (25.3)	289.0 (32.0)	356.7 (17.7)	336.3 (31.4)	302.5 (19.7)	338.2 (36.5)	**<0.0001**	**<0.0001**	**0.0026**	0.2225

**†Final blood and urine biochemistries**. ANOVA p-values for the overall model (P_Model_), main effect of age (P_Age_), main effect of diet (P_Diet_), and interaction of age and diet (P_AxD_) are shown, and means and (SEM) are shown for each group. Plasma phosphorus values were higher in 10 week olds vs 20 and 30 weeks. An age x diet interaction for plasma calcium was driven by an increase at 10 weeks on the low phosphorus diet. Plasma creatinine was higher in 10 week olds but had a marginal interaction with diet. Urinary creatinine increased at each age group. Blood hematocrit was not different between age or diet groups. Creatinine clearance was lower at 10 weeks vs 20 and 30 weeks. Plasma BUN progressively declined with age. There was a significant age x diet interaction for plasma PTH were low phosphorus resulted in lower PTH compared to normal and high phosphorus, but the magnitude was greatest at 10 weeks. iFGF23 was lower in the low phosphorus group compared to normal and high, and lower at 10 weeks compared to 20 weeks. cFGF23 was lower in the low phosphorus group compared to normal and high phosphorus. Plasma 1,25D was higher at 10 weeks compared to 20 and 30 weeks, and higher on the low phosphorus compared to normal phosphorus diet. LP = low phosphorus diet, NP = normal phosphorus diet, HP = high phosphorus diet.

^a^ n = 4 excluded for insufficient plasma.

^b^ n = 12 excluded for insufficient sample.

^c^ n = 2 excluded for insufficient plasma.

^d^ n = 3 excluded as unphysiologic outliers (near zero).

^e^ n = 6 excluded for insufficient plasma.

**Fig 1:**
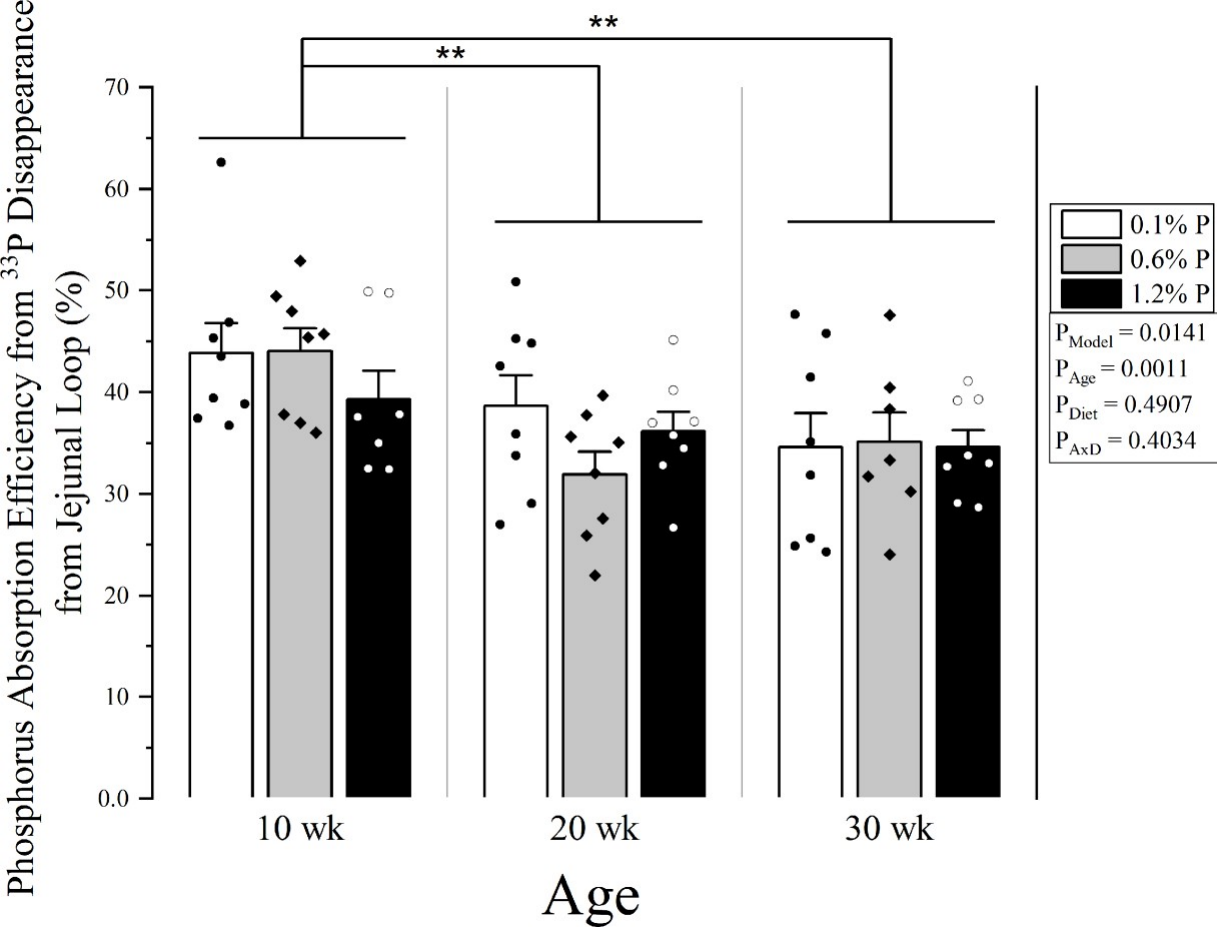
Percent jejunal phosphorus absorption efficiency by age and dietary phosphorus intake level. Phosphorus absorption efficiency was calculated as 1-(^33^P activity remaining in jejunal loop)/(Total ^33^P activity in dose) after 30 minutes post ^33^P injection into the jejunal loop. Means and standard error bars are shown for each group. Low phosphorus diet (0.1%) is shown in white bars and black dots; normal phosphorus diet (0.6%) is shown in grey with black diamonds; and high phosphorus diet (1.2%) is shown in black with white circles. ANOVA p-values for the overall model (P_Model_), main effect of age (P_Age_), main effect of diet (P_Diet_), and interaction of age and diet (P_AxD_) are shown. There was a main effect for age where 10 week old rats had higher phosphorus absorption compared to both 20 and 30 week olds, but there was no significant effect of dietary phosphorus intake level and no significant age x diet interaction. ** p < 0.01. n = 2 excluded, 1 for mishandling loop, and 1 unphysiologic (counts ~150 fold lower than expected).

**Fig 2:**
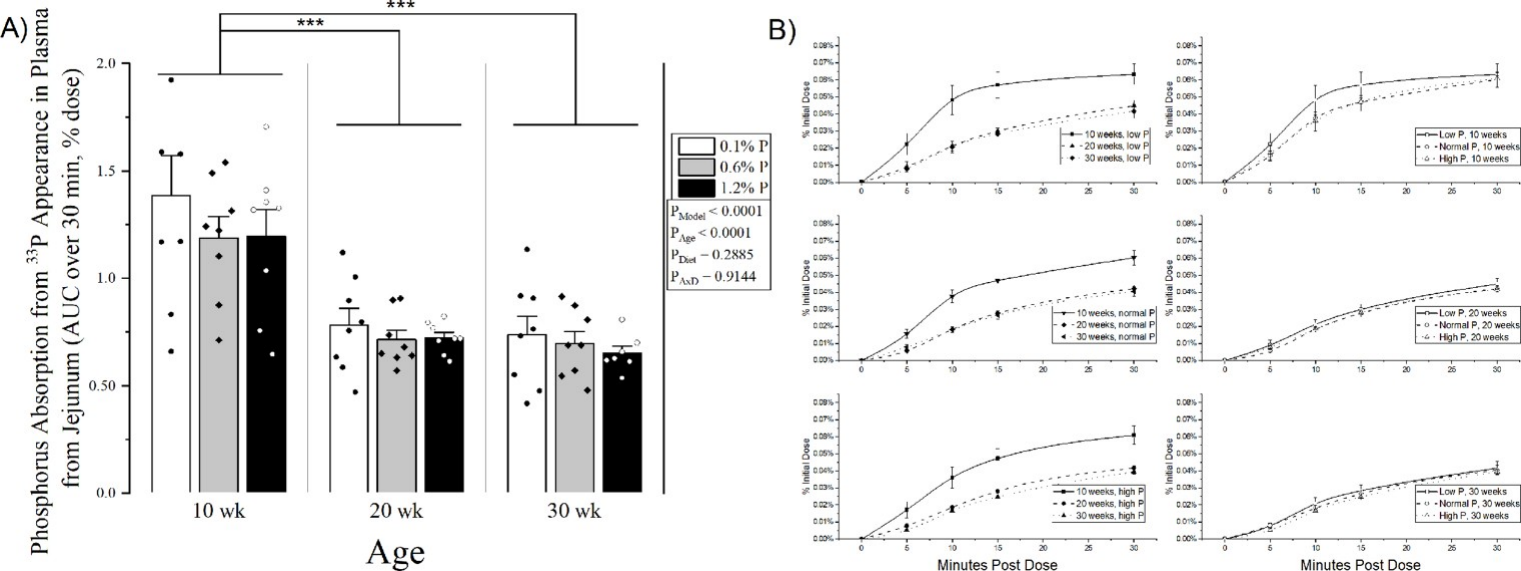
**A) Jejunal phosphorus absorption determined by appearance of** ^**33**^**P in plasma over 30 minutes (AUC).** Means and standard error bars are shown for each group. Low phosphorus diet (0.1%) is shown in white bars and black dots; normal phosphorus diet (0.6%) is shown in grey with black diamonds; and high phosphorus diet (1.2%) is shown in black with white circles. ANOVA p-values for the overall model (P_Model_), main effect of age (P_Age_), main effect of diet (P_Diet_), and interaction of age and diet (P_AxD_) are shown. **B) Jejunal phosphorus absorption determined by appearance of** ^**33**^**P in plasma over 30 minutes (time series).** Each diet group (left) and each age group are compared (right). Results at 30 minutes were similar to AUC. Absorption was calculated as percent of total ^33^P activity in the initial dose. There was a main effect for age where 10 week old rats had higher phosphorus absorption compared to both 20 and 30 week olds, but there was no significant effect of dietary phosphorus intake level and no significant age x diet interaction. *** p < 0.0001. n = 1 excluded for missing last 2 timepoints.

Percent intestinal phosphorus absorption efficiency (percent of dose), as assessed by disappearance from the intestinal loop at 30 minutes, was higher in 10-week-olds compared to both 20- and 30-week-olds (42.5 ± 0.02% vs 35.6 ± 0.01% and 34.7 0.02%, p < 0.01 for both), but 20- and 30-week-olds were similar (**Fig 1**). Correspondingly, the plasma ^33^P activity (percent of dose) AUC over 30 minutes was higher in 10 weeks-olds compared to both 20- and 30- week-olds (1.3 ± 0.08% vs 0.7 ± 0.03% and 0.7 ± 0.04%, p < 0.0001 for both) (**Fig 2**) There was no effect of dietary phosphorus level on either absorption measure (loop: p = 0.4907; plasma AUC: p = 0.2585), nor any significant age x diet interaction (loop: p = 0.4034; plasma AUC: p = 0.9986). Similar results were observed when plasma ^33^P activity at only the final 30-minute time point was analyzed (p = 0.6742 for diet main effect, p < 0.0001 for 10 week-olds vs 20- and 30-week-olds).

**Fig 3:**
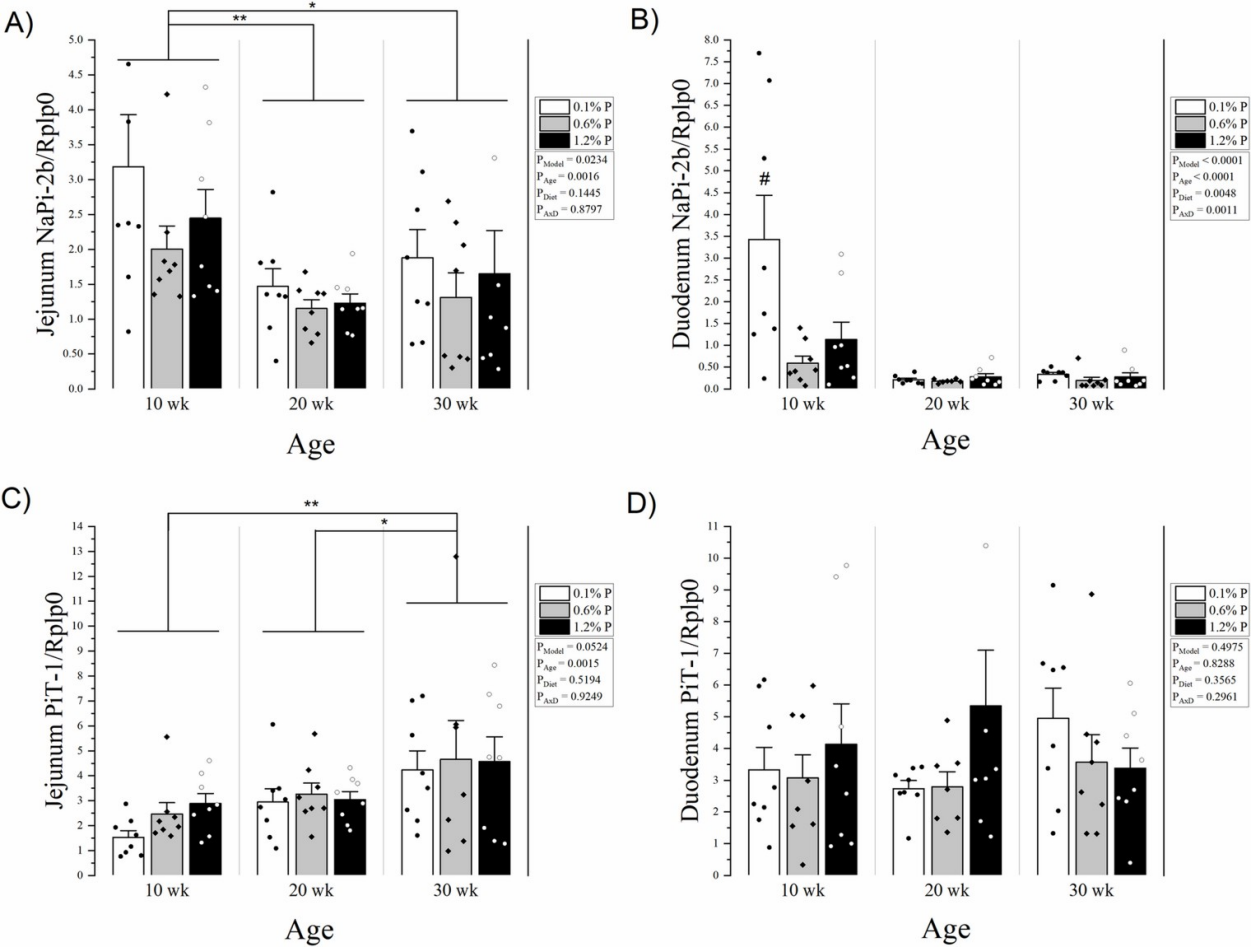
**A) Jejunal NaPi-2b mRNA expression by age and dietary phosphorus intake level.** There was a main effect for age where 10 week old rats had higher NaPi-2b expression compared to both 20 and 30 week olds, but there was no significant effect of dietary phosphorus intake level and no significant age x diet interaction. **B) Duodenal NaPi-2b mRNA expression by age and dietary phosphorus intake level.** There was a significant interaction driven by a higher expression in 10 week rats on a low phosphorus diet. **C) Jejunal PiT-1 mRNA expression by age and dietary phosphorus intake level.** The overall model approached significance, with a main effect for age where 30 week old rats had higher PiT-1 expression compared to both 20 and 10 week olds, but there was no significant effect of dietary phosphorus intake level and no significant age x diet interaction. **D) Duodenal PiT-1 mRNA expression by age and dietary phosphorus intake level.** There were no differences in age, diet, or interaction. Expression was calculated relative to Rplp0. Means and standard error bars are shown for each group. Low phosphorus diet (0.1%) is shown in white bars and black dots; normal phosphorus diet (0.6%) is shown in grey with black diamonds; and high phosphorus diet (1.2%) is shown in black with white circles. ANOVA p-values for the overall model (P_Model_), main effect of age (P_Age_), main effect of diet (P_Diet_), and interaction of age and diet (P_AxD_) are shown. * p < 0.05, ** p < 0.01, # p < 0.01 vs all other groups. n = 1 duodenum NaPi-2b and PiT-1 excluded for missing sample.

Similar to the jejunal ligated loop absorption results, jejunal NaPi-2b mRNA was higher in 10-week-olds compared to both 20- and 30-week-olds (p = 0.0016 and p = 0.0245, respectively) (**Fig 3A**). In the duodenum, there was an age x diet interaction (p = 0.0011) driven by higher NaPi-2b mRNA levels in 10-week-olds on the low phosphorus diet compared to all other groups (**Fig 3B**). Jejunal PiT1 mRNA tended to be higher at 30-weeks, although the overall model did not reach statistical significance (p = 0.0524) (**Fig 3C**). Duodenal PiT1 expression was not significantly affected by diet or age (overall model p = 0.4975) (**Fig 3D**).

**Fig 4:**
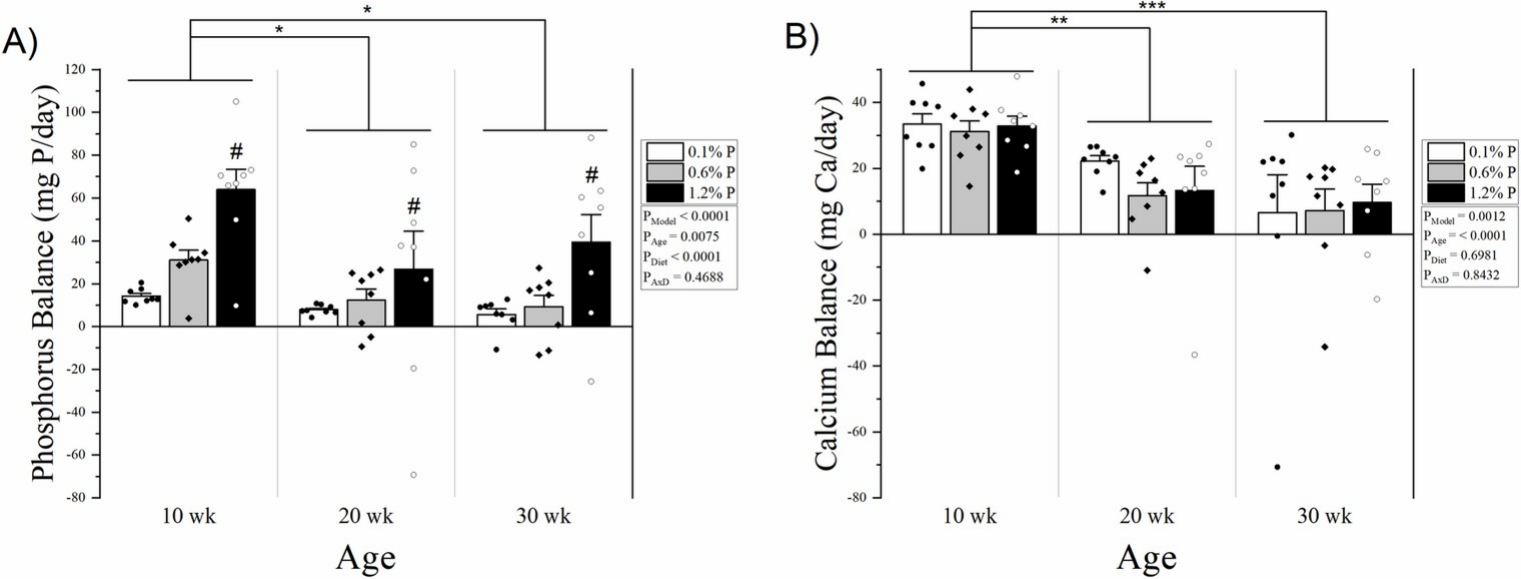
**A) Phosphorus balance by age and dietary phosphorus intake level.** There was a main effect for age where 10 week old rats had higher phosphorus balance compared to both 20 and 30 week olds, and the high phosphorus diet group was higher than normal and low phosphorus, with no significant age x diet interaction. **B) Calcium balance by age and dietary phosphorus intake level.** There was a main effect for age where 10 week old rats had higher calcium balance compared to both 20 and 30 week olds, but there was no significant effect of dietary phosphorus intake level and no significant age x diet interaction. Balance for each mineral was calculated as intake–fecal + urine. Means and standard error bars are shown for each group. Low phosphorus diet (0.1%) is shown in white bars and black dots; normal phosphorus diet (0.6%) is shown in grey with black diamonds; and high phosphorus diet (1.2%) is shown in black with white circles. ANOVA p-values for the overall model (P_Model_), main effect of age (P_Age_), main effect of diet (P_Diet_), and interaction of age and diet (P_AxD_) are shown. * p < 0.05, ** p < 0.01, *** p < 0.0001.

**Fig 5:**
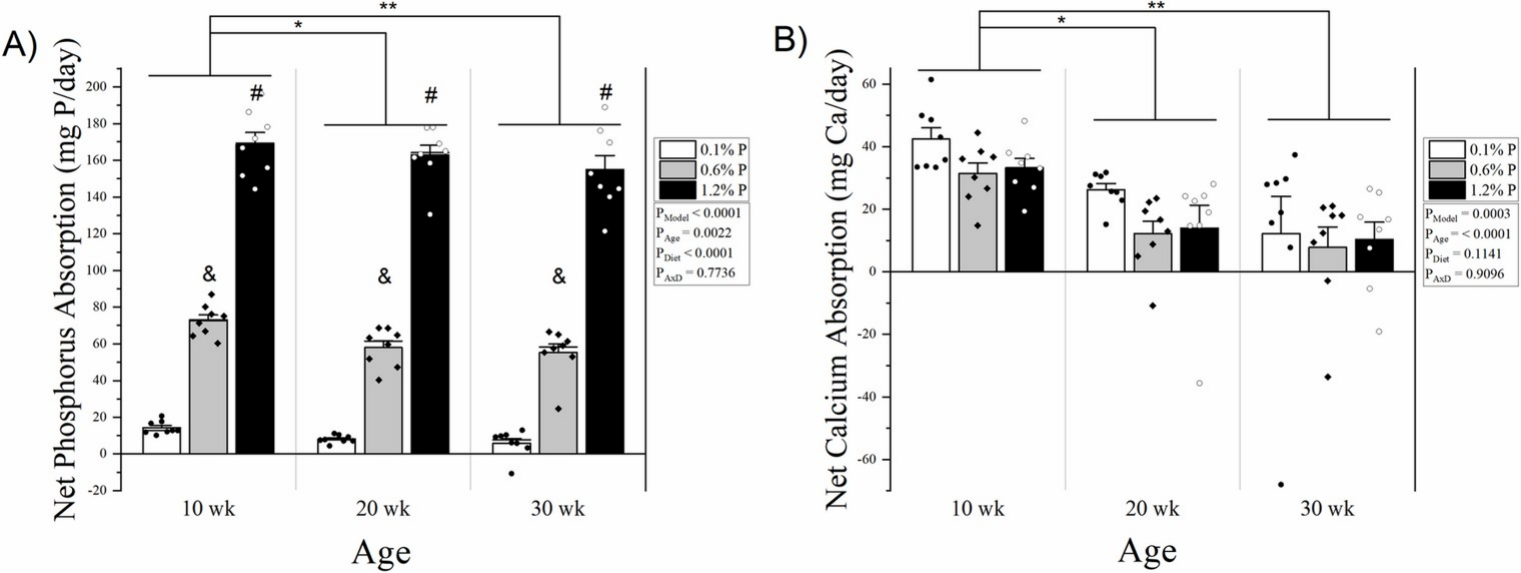
**A) Net phosphorus absorption by age and dietary phosphorus intake level.** There was a main effect for age where 10 week old rats had higher net phosphorus absorption compared to both 20 and 30 week olds, and net absorption increased with each phosphorus level in the diet, with no significant age x diet interaction. **B) Net calcium absorption by age and dietary phosphorus intake level.** There was a main effect for age where 10 week old rats had higher calcium balance compared to both 20 and 30 week olds, but there was no significant effect of dietary phosphorus intake level and no significant age x diet interaction. Net absorption for each mineral was calculated as intake–fecal. Means and standard error bars are shown for each group. Low phosphorus diet (0.1%) is shown in white bars and black dots; normal phosphorus diet (0.6%) is shown in grey with black diamonds; and high phosphorus diet (1.2%) is shown in black with white circles. ANOVA p-values for the overall model (P_Model_), main effect of age (P_Age_), main effect of diet (P_Diet_), and interaction of age and diet (P_AxD_) are shown. * p < 0.05, ** p < 0.01, # p < 0.0001 vs normal and low phosphorus, & p < 0.0001 vs high and low phosphorus.

Phosphorus balance was higher in 10-week-olds compared with both 20- and 30-week-olds, and not different between 20- and 30-week-olds (36.4 ± 5.5 mg/day vs 15.7 ± 6.1 mg/day and 18.1 ± 5.5 mg/day, p = 0.0113 and 0.0286, respectively) **(Fig 4A)**. There was a significant effect for diet on phosphorus balance, where HP was higher than NP and LP (43.4 ± 8.2 mg/day vs 17.5 ± 3.4 mg/day and 9.2 ± 1.2 mg/day, p = 0.0012 and p < 0.0001, respectively). For net phosphorus absorption, there was a clear dose-response effect of diet (LP: 9.4 ± 1.2 mg/day, NP: 62.0 ± 2.7 mg/day, HP: 162.2 ± 3.8 mg/day, p < 0.0001 for all comparisons), and 10-week-olds had higher net phosphorus absorption than 20- and 30-week old rats (85.3 ± 13.5 mg/day vs 76.4 ± 13.6 mg/day and 72.0 ± 13.3 mg/day, p = 0.0469 and p < 0.0017, respectively), but 20- and 30-week-olds were not different **(Fig 5A)**. Calcium balance was also higher in 10-week-old rats compared with both 20- and 30-week but not between 20- and 30-week-olds (32.5 ± 1.7 mg/day vs 15.8 ± 2.9 mg/day and 7.8 ± 4.6 mg/day, p = 0.0024 and p < 0.0001, respectively **(Fig 4B)**. Net calcium absorption was similarly higher in 10-week rats (35.7 ± 2.1 mg/day vs 17.5 ± 3.0 mg/day and 10.1 ± 4.7 mg/day, p = 0.0012 and p < 0.0001) **(Fig 5B)**. Phosphorus in the diet had no effect on calcium balance nor net absorption of calcium (p = 0.6981 and p = 0.1141 for main effect) **(Figs 4B and 5B)**.

## Discussion

In this study, we found higher intestinal phosphorus absorption at 10-weeks of age compared to 20- and 30-weeks as assessed by the ligated loop technique in both appearance of ^33^P in plasma and disappearance of ^33^P from the intestinal loop. This interpretation is supported by the more positive net phosphorus absorption from metabolic balance and more positive overall phosphorus balance, higher plasma phosphorus, and higher NaPi-2b mRNA expression in the 10-week rats vs the 20- and 30-week-olds. The lack of differences between 20- and 30-week-old rats is likely due to less metabolic demand of bone for phosphorus. The increased phosphorus absorption and positive phosphorus balance corresponded to higher serum phosphorus levels at a younger age, similar to that in humans [23, 24]. Further, there was lower PTH and FGF23 levels and higher 1,25D levels at the younger age, suggesting that hormonal regulation decreases renal phosphorus excretion via decreased PTH and FGF23, and increases intestinal absorption via vitamin D [7]. The elevation at 10-weeks likely reflects the increased requirement of phosphorus for growth at this age [25] but how these hormonal changes are stimulated during growth is not completely understood.

Other studies that have observed differences in sodium-dependent BBMV phosphate uptake between post-weaning and adult rodents have studied even older rats and still found no differences compared with younger adult rats. Armbrecht [26] compared 8–12 week-old (“young’) vs 48-46-week-old (“adult”) and 88-96-week-old (“old”) Fisher 344 rats, and observed differences between the young vs adult and old rats, but no difference between the adult and old rats. This suggests that sodium-*dependent* absorption decreases after growth then plateaus for the adult lifespan. Borowitz and Ghishan [27] also showed an age-dependent decrease in jejunal sodium-*independent* BBMV phosphate uptake from 2 to 6 weeks-of-age, albeit smaller than sodium-dependent reductions. The changes to sodium-*independent* phosphate transport in later stages of aging should be further evaluated, as this may contribute to a greater portion of total phosphorus absorption with aging. In the pre-weaning mouse, an age-dependent decrease in NaPi-2b occurs from 14 days to 21 days to 8 weeks, and then remains the same until 8–9 months (32–36 weeks) [28]. In rats, there is an age-dependent decrease in NaPi-2b gene expression from 2 weeks to 3 weeks to 6 weeks and 95–100 days (13.5–14.3 weeks), and a reduction in BBMV uptake between 2 weeks and 95–100 days [29]. Future work is necessary to characterize changes in absorption during this transition using more physiologic absorption techniques.

Importantly, we did not find a difference by dietary phosphorus level on intestinal phosphorus absorption assessed by jejunal ligated loop. We used the same low phosphorus diet (0.1%) that has been shown to increase jejunal NaPi-2b expression and sodium-dependent phosphate uptake in rats *in vitro* [11, 12], but don’t see that translate in our study to an effect on the *in situ* ligated loop technique using a transport buffer phosphate concentration that would be expected to favor sodium-dependent transport [30]. The sodium-dependent, transcellular pathway predominates at low luminal phosphate concentrations, whereas the sodium-independent, paracellular pathway will contribute more at high luminal phosphate concentrations [30–32]. The latter was reflected in our results by the stepwise increase in net phosphorus absorption corresponding to the amount of phosphorus in the diet. NaPi-2b is currently understood to be the main sodium-dependent intestinal phosphate transporter. It shares homology to the renal NaPi-2a/c transporters [33, 34] and the type III sodium-phosphate co-transporters, PiT-1 and PiT-2, that are considered to play more minor roles in absorption [33–37]. NaPi-2b is estimated to contribute >90% of sodium-dependent transport based on a mouse NaPi-2b knockout model [36], with PiT-1 and PiT-2 believed to contribute <10% of sodium-dependent transport [37]. We did not measure PiT-2 because its expression in the intestine is very low [38] and others have found that PiT-2 mRNA does not change in response to dietary phosphorus restriction [10]. Interestingly, recent evidence suggests that additional transporters may be yet undiscovered [12]. Our data question the importance of sodium-dependent transcellular phosphate transport in the presence of a liberal consumption of dietary phosphorus which would favor the sodium-independent pathway, characteristic of the American diet [39]. Other rodent and pig studies have consistently found increases in *in vitro* intestinal BBMV phosphate uptake after a low phosphorus diet [9–14, 16, 17, 40], and while studies that measure NaPi-2b protein expression consistently find increases [11, 12, 14, 16, 18, 40, 41], NaPi-2b mRNA expression only increases in some [14, 16, 41], but not all studies [10, 15, 20]. A limitation in our study is a lack of transporter protein expression. In contrast, and similar to our findings, the previous studies in rodents using the more physiologic *in situ* ligated loop technique for effects of low phosphorus diets have had conflicting results [19, 20]. Rizzoli et al. [19] observed higher duodenal phosphate transport after a 15-minute ligated loop on a low phosphorus (0.2%) diet in normal female rats compared to a normal phosphorus (0.8%) diet after 16 days of feeding, but not after only 8 days, and higher duodenal phosphate transport with a normal phosphorus (0.8%) diet compared to a high phosphorus (1.8%) diet after 8 days. However, these results were split between two separate experiments with different concentrations of phosphate in the absorption buffers (5 mM and 2 mM), making it difficult to compare low vs high phosphorus intake. More recently, Marks et al. [20] tested the effects of very low phosphorus (0.02%) versus normal phosphorus (0.52%) diet on jejunal phosphate transport efficiency in 5/6^th^ nephrectomized male rats with a 30-minute jejunal ligated loop. No effect of diet on phosphorus absorption was observed, nor any change in jejunal or duodenal NaPi-2b mRNA expression. Together, the results of our study which utilized both loop disappearance and plasma ^33^P counts, and the other *in situ* studies, suggest that conclusions on the effects of low phosphorus diets on phosphorus absorption from studies utilizing *in vitro* and *ex vivo* uptake/transport or flux techniques which utilize isolated intestinal segments or BBMV may not appropriately reflect physiologic conditions affecting phosphorus absorption. Thus, there is need for additional studies that employ more physiological methods of phosphorus absorption assessment. Our study was limited to male rats, so we were unable to determine if sex-differences exist for age and dietary phosphorus intake level effects on intestinal phosphorus absorption by the ligated loop technique. To our knowledge, ours is the first study to utilize the ligated loop method on normal male rats to test this question.

*In vitro* techniques may fail to replicate physiologic *in vivo* techniques for a number of reasons. First, even when using a low transport buffer phosphate concentrations (0.1 mM KH_2_PO_4_) [31] close to the Km of NaPi-2b [42], residual luminal phosphate makes it such that sodium-dependent transport is contributing a smaller proportion to total phosphate transport and mask effects of interventions on the sodium-dependent transport mechanism. Luminal phosphate concentration *in vivo* is ~1.5–40 mM in the proximal intestine depending on measurement technique [31, 43], which may contribute to a higher passive transport than *in vitro* techniques. Thus, estimating this contribution with the ligated loop is challenging. Marks and colleagues assessed the proportion of sodium-dependent absorption with the ligated loop in rats at 32 ± 8% vs 73 ± 5% using the everted sleeve with the phosphate concentration that we utilized [31]. It is likely that excreted luminal sodium during the test results in sodium-dependent transport when injecting a solution without sodium, and indeed measurable sodium was present at the end of the test. Therefore, we assume that 32% sodium-dependency is a low-end estimate using this technique. While it is a limitation that we didn’t attempt to measure changes in sodium-independent absorption with respect to dietary phosphorus and age, sodium-independent uptake is not regulated by dietary phosphorus [10, 13, 40, 44]. Additional factors such as a disconnection to local blood flow and nerves or transmural potential differences may explain *in situ*/*in vivo* differences with *in vitro/ex vivo* techniques [31]. It is also possible that other intestinal segments would respond to the two factors we tested. However our interest in studying the jejunum as regional specific regulation of phosphorus absorption was because 1) NaPi-2b is expressed highest in the jejunum in rats [45], 2) some studies show that NaPi-2b mRNA and protein only respond to chronic phosphorus restriction in the jejunum but not duodenum [38], and only uptake increased in the jejunum [38]. Further, infusion of Matrix Extracellular Phosphoglycoprotein in rats led to a selective change in absorption rate in the jejunum but not duodenum, suggesting that the jejunum may be most responsive to other factors as well [46]. However, Rizzoli and colleagues showed an increased absorption efficiency in the duodenal segment in rats with the loop [19], whereas others have showed age related decreases in uptake with age in both the jejunum and duodenum [26]. Additional research is needed to clarify the relative importance of capacity of the duodenum to adapt to various factors.

In conclusion, in the present study we examined the interaction of age and diet on intestinal phosphorus absorption in healthy male Sprague Dawley rats with the *in situ* ligated loop technique. Moderate phosphorus restriction (0.1%) did not affect phosphate absorption with this technique, but absorption was higher in younger (10-week) rats with higher positive phosphorus balance.

## Supporting information

S1 File Quench curve preparation. (DOCX)

S2 File Primary data. (XLSX)
