# Supplementary material for: Effect of dietary phosphorus intake and age on intestinal phosphorus absorption efficiency and phosphorus balance in male rats
Source: PLoS One. 2018 Nov 19;13(11):e0207601. doi: 10.1371/journal.pone.0207601 (PMC6242370; doi:10.1371/journal.pone.0207601)

**Supplemental Information for 33P Quench Curves**

Plasma and intestinal ligated loop samples and standards were counted for ^33^P radioactivity by liquid scintillation. Various constituents of these samples can cause both chemical and color quenching that artificially lowers counts per minute (CPM) detected by the liquid scintillation counter. Therefore, two quench curves were developed to correct for sample quench using the same scintillation cocktails as used for the samples. A standard quenching agent, nitromethane, was used to create the quench curve for use with the plasma samples, but in the case of the digested ligated loops, the sources of potential quench in the samples were simulated to create the quench curve. These quench curves were prepared based on methods described by Thomas (2014).^(1)^

***Quench curve for plasma counting***

***Preparation of the quench curve***

Thirteen replicate scintillation vials containing 15mL EcoLite scintillation cocktail (MP Biomedicals, Santa Ana, CA) and ~250,000 CPM ^33^P (200 μL 0.5 μCi/mL ^33^P-orthophosphate (Perkin Elmer, Waltham, MA) in deionized water) were prepared. Vials were counted on a Tri-Carb 2910 TR Liquid Scintillation Counter (Perkin Elmer, Waltham, MA) in a counting energy window of 0-300 keV for 10 minutes per vial (energy of ^33^P: 76.4 keV average, 248.5 keV maximum) and CPM values obtained. The two vials that deviated the most from the mean CPM were discarded. The remaining 11 vials had mean radioactivity counts of 258,447 CPM and CV = 0.3%. These 11 vials were used to prepare a quench curve using nitromethane (Fisher Scientific, Hampton, NH) as a quenching agent in increasing amounts (**Table S1**). The quench parameter tSIE/AEC is determined from an external ^133^Ba gamma source within the instrument, which is described in further detail in Thomas (2014).^(1)^

***Results***

The quench values (tSIE/AEC) and counting efficiencies (%) are presented in **Table S1**, and the quench curve is plotted in **Figure S1**. Unquenched ^33^P (200 μL 0.5 μCi/mL solution in 15 mL scintillation cocktail) had a 98% counting efficiency. Counting efficiency was above 95% until the quench parameter tSIE/AEC reached ~200 (vial #7 =197 tSIE/AEC). Counting efficiency remained relatively high (above 70%) even with very high quench values seen in vials #9 and #10, and was only reduced to 59% in the most quenched (tSIE/AEC = 37) vial #11. Plasma samples analyzed in the present rat study had quench values in the range of ~470-490 tSIE/AEC which placed them on the quench curve at > 98% counting efficiency. Thus, correcting for counting efficiency only resulted in minor changes in radioactivity values of the rat plasma samples. Statistical analyses of data before and after quench curve corrections produced similar results for ANOVA model effects and group differences, but quench correction slightly increased group means.

***Quench curve for intestinal ligated loop counting***

***Preparation of the quench curve***

The present rat study utilized an *in situ* jejunal ligated loop method for phosphorus absorption assessment. Percent phosphorus absorption efficiency was calculated as:

1 − (^33^P activity remaining in digested loop) / (Total ^33^P activity in 0.5mL dose) ∙ 100. To determine the ^33^P activity remaining in digested loop, the ~5cm excised intestinal segment was digested in 6 mL Soluene 350 (PerkinElmer, Waltham, MA) in a 45^o^C oven, then divided into two scintillation vials and the color was lightened with the addition of 0.6mL of 30% hydrogen peroxide into each of the two vials for the purpose of reducing color quench. Total ^33^P activity remaining in the digested loop was calculated by adding the counts from the two vials with the split sample. Therefore, an appropriate quench curve for the digested intestinal loops would simulate the constituents in the vials that are potential sources of quench. These factors include: intestinal tissue, thread from ligatures used to tie off the loop segment, absorption buffer solution injected into the ligated loop during the absorption test.

A range of these factors below and above the actual amounts and volumes utilized in the study were used in creating a quench curve. Intestinal tissue, thread, and solutes were added to nine scintillation vials (see lengths and volumes in **Table S2**) and left to digest for 3 days in an oven at 45^o^C. Approximately 85,000 CPM ^33^P (50 μL 0.8 μCi/mL ^33^P-orthophosphate (Perkin Elmer, Waltham, MA) in deionized water) was added into each vial. Hydrogen peroxide and Hionic Fluor scintillation cocktail (PerkinElmer, Waltham, MA) were added to each vial in amounts shown in **Table S2**. Vials were counted on a Tri-Carb 2910 TR Liquid Scintillation Counter in an open energy window of 0-2000 keV for 30 minutes per vial. The quench parameter tSIE/AEC was obtained from the counter output.

***Results***

The quench values (tSIE/AEC) and counting efficiencies (%) are presented in **Table S2**, and the quench curve is plotted in **Figure S2**. Unquenched ^33^P (50 μL 0.8 μCi/mL solution in 20 mL scintillation cocktail) had > 99% counting efficiency. Counting efficiency declined to 89% with the addition of tissue, thread, and solutes. Using the same amounts of these factors as the used for the actual study samples (“normal conditions”, vials 4 and 5), counting efficiency was 87% with a quench value of 196 tSIE/AEC. As shown in **Figure S2**, two vials (open blue circles on figure, vials 6 and 8) were excluded because their counting efficiencies did not follow the expected trend based on the curve established by the adjacent vials.

Intestinal loop samples analyzed in the current study had quench values in the range of ~150-230 tSIE/AEC, which placed them on the quench curve ~85-88% efficiency. Thus, correcting for counting efficiency based on the quench curve was important for reporting accurate intestinal phosphorus absorption values. Statistical analyses of data before and after quench curve corrections produced similar results for ANOVA model effects and group differences, but quench correction increased group means.

**Conclusions**

Counting efficiency by liquid scintillation counting is relatively high for ^33^P for both plasma and digested intestinal ligated loop segments. However, the quench curves developed for the two types of samples were not interchangeable when comparing the tSIE/AEC values and associated % counting efficiencies on each curve (e.g. on the Ecolite/nitromethane curve, a quench value of 197 tSIE/AEC had a counting efficiency of 95%, but on the Hionic Fluor/Loop curve, a quench values of 195 tSIE/AEC had a counting efficiency of only 87%. Therefore, not only would unadjusted ^33^P activity in digested ligated loops underestimate actual radioactivity in a sample, utilizing the wrong quench curve could also produce an erroneous result. This underscores the importance of utilizing a quench curve that simulates as closely as possible the conditions of the samples to be counted.^(1)^

**Reference**

1. Thomson J. Use and Preparation of Quench Curves in Liquid Scintillation Counting. 2014.

**Table S1. ^33^P quench curve with EcoLite scintillation cocktail for plasma counting**

| Vial # | Amount (μL) of nitromethane | tSIE/AEC | Count Efficiency (%) |
| --- | --- | --- | --- |
| 1 | 0 | 468.63 | 98.22 |
| 2 | 5 | 435.48 | 99.83 |
| 3 | 10 | 401.87 | 98.84 |
| 4 | 15 | 378.68 | 98.63 |
| 5 | 26 | 325.32 | 98.66 |
| 6 | 45 | 258.69 | 96.55 |
| 7 | 70 | 197.87 | 95.58 |
| 8 | 110 | 134.92 | 90.84 |
| 9 | 150 | 97.21 | 86.69 |
| 10 | 230 | 55.73 | 73.77 |
| 11 | 310 | 37.89 | 59.63 |

**Figure S1. Quench curve of ^33^P in EcoLite scintillation cocktail for plasma counting**


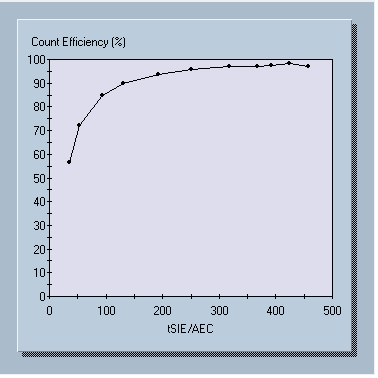


**Table S2. ^33^P quench curve with Hionic Fluor scintillation cocktail for loop counting**

| Vial # | Intestinal Segment Length (cm) | Ligature  Thread (cm) | Soluene 350 vol. (mL) | Buffer vol. (10x dil.) (μL) | Hionic Fluor vol. (mL) | Hydrogen peroxide (mL) | tSIE/AEC | Count Efficiency (%) |
| --- | --- | --- | --- | --- | --- | --- | --- | --- |
| 1 | 0 | 0 | 0 | 0 | 20 | 0 | 315.13 | 99.78 |
| 2 | 1 | 0.5 | 1 | 50 | 19 | 0.1 | 278.96 | 89.30 |
| 3 | 1.5 | 1 | 1.5 | 50 | 18.5 | 0.2 | 272.47 | 89.05 |
| 4 (“normal conditions”) | 2.5 | 1.5 | 3 | 200 | 17 | 0.6 | 195.98 | 87.48 |
| 5 (“normal conditions”) | 2.5 | 1.5 | 3 | 200 | 17 | 0.6 | 195.85 | 87.25 |
| 6 | 3 | 2 | 3.5 | 250 | 16.5 | 0.7 | 174.52 | 62.86 |
| 7 | 3.5 | 2 | 3.5 | 250 | 16.5 | 0.8 | 169.07 | 85.96 |
| 8 | 4 | 3 | 4 | 300 | 16 | 0.8 | 153.06 | 80.22 |
| 9 | 4.5 | 3.5 | 4 | 350 | 16 | 0.8 | 145.43 | 84.24 |

Vials deemed outliers are indicated here in red text.

**Figure S2. Quench curve of ^33^P in Hionic Fluor scintillation cocktail for loop counting**
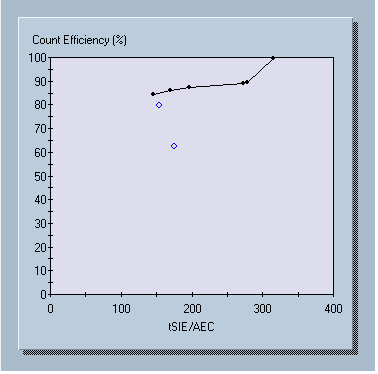

Supplement: S1 File — (DOCX) [file pone.0207601.s001.docx]
